# Electroencephalogram rhythmic and arrhythmic spectral components and functional connectivity at resting state may predict the development of synucleinopathies in idiopathic rapid eye movement sleep behavior disorder

**DOI:** 10.1093/sleep/zsae074

**Published:** 2024-03-18

**Authors:** Jimmy Hernandez, Jean-Marc Lina, Jonathan Dubé, Alexandre Lafrenière, Jean-François Gagnon, Jacques-Yves Montplaisir, Ronald B Postuma, Julie Carrier

**Affiliations:** Center for Advanced Research in Sleep Medicine, Research center, CIUSSS du Nord de l’Île-de-Montréal, Montreal, QC, Canada; Department of Neuroscience, Université de Montréal, Montreal, QC, Canada; Center for Advanced Research in Sleep Medicine, Research center, CIUSSS du Nord de l’Île-de-Montréal, Montreal, QC, Canada; Department of electrical engineering, École de technologie supérieure, Montreal, QC, Canada; Center for Advanced Research in Sleep Medicine, Research center, CIUSSS du Nord de l’Île-de-Montréal, Montreal, QC, Canada; Department of Psychology, Université de Montréal, Montreal, QC, Canada; Center for Advanced Research in Sleep Medicine, Research center, CIUSSS du Nord de l’Île-de-Montréal, Montreal, QC, Canada; Department of Psychology, Université de Montréal, Montreal, QC, Canada; Center for Advanced Research in Sleep Medicine, Research center, CIUSSS du Nord de l’Île-de-Montréal, Montreal, QC, Canada; Department of Psychology, Université du Québec à Montréal, Montreal, QC, Canada; Center for Advanced Research in Sleep Medicine, Research center, CIUSSS du Nord de l’Île-de-Montréal, Montreal, QC, Canada; Department of psychiatry, Université de Montréal, Montreal, QC, Canada; Center for Advanced Research in Sleep Medicine, Research center, CIUSSS du Nord de l’Île-de-Montréal, Montreal, QC, Canada; Department of Neurology and Neurosurgery, Montreal Neurological Institute, Montreal, QC, Canada; Center for Advanced Research in Sleep Medicine, Research center, CIUSSS du Nord de l’Île-de-Montréal, Montreal, QC, Canada; Department of Psychology, Université de Montréal, Montreal, QC, Canada

**Keywords:** idiopathic/isolated REM sleep behavior disorder, synucleinopathy, Parkinson’s disease, dementia with Lewy bodies, resting-state EEG, spectral power, arrhythmic component, functional connectivity

## Abstract

**Study Objectives:**

Idiopathic/isolated rapid eye movement-sleep behavior disorder (iRBD) often precedes the onset of synucleinopathies. Here, we investigated whether baseline resting-state EEG advanced spectral power and functional connectivity differed between iRBD patients who converted towards a synucleinopathy at follow-up and those who did not.

**Methods:**

Eighty-one participants with iRBD (66.89 ± 6.91 years) underwent a baseline resting-state EEG recording, a neuropsychological assessment, and a neurological examination. We estimated EEG power spectral density using standard analyses and derived spectral estimates of rhythmic and arrhythmic components. Global and pairwise EEG functional connectivity analyses were computed using the weighted phase-lag index (wPLI). Pixel-based permutation tests were used to compare groups.

**Results:**

After a mean follow-up of 5.01 ± 2.76 years, 34 patients were diagnosed with a synucleinopathy (67.81 ± 7.34 years) and 47 remained disease-free (65.53 ± 7.09 years). Among patients who converted, 22 were diagnosed with Parkinson’s disease and 12 with dementia with Lewy bodies. As compared to patients who did not convert, patients who converted exhibited at baseline higher relative theta standard power, steeper slopes of the arrhythmic component and higher theta rhythmic power mostly in occipital regions. Furthermore, patients who converted showed higher beta global wPLI but lower alpha wPLI between left temporal and occipital regions.

**Conclusions:**

Analyses of resting-state EEG rhythmic and arrhythmic components and functional connectivity suggest an imbalanced excitatory-to-inhibitory activity within large-scale networks, which is associated with later development of a synucleinopathy in patients with iRBD.

## Graphical Abstract

**Graphical Abstract:**
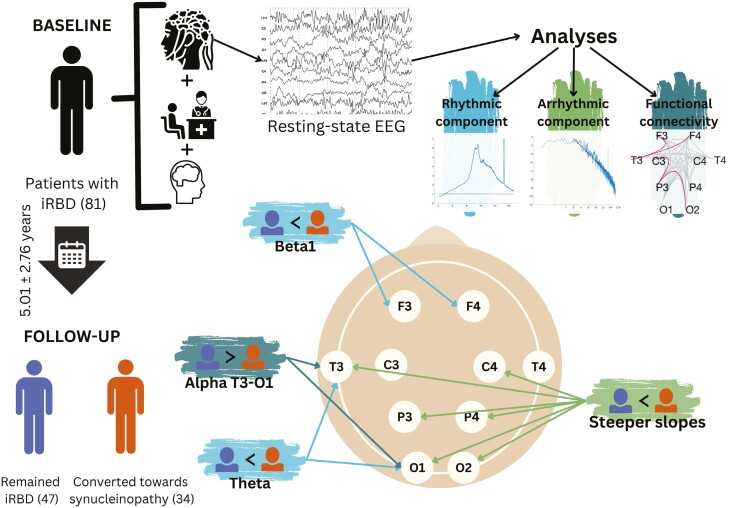

**Statement of Significance**

Recent studies have highlighted alterations in functional connectivity and both rhythmic and arrhythmic components of resting-state EEG in patients with Parkinson’s disease or dementia with Lewy bodies. This research revealed that these changes can be detected in individuals with iRBD years before they receive a clinical synucleinopathy diagnosis. Notably, iRBD patients who later developed synucleinopathy exhibited steeper slopes of the arrhythmic component, higher theta rhythmic power in posterior cortical regions, increased beta global functional connectivity, but reduced alpha functional connectivity between the left temporal and occipital regions during baseline assessments. These findings suggest disruptions in the inhibitory/excitatory balance and functional integration within large-scale cerebral networks in iRBD patients at high risk of short-term phenoconversion.

## Introduction

Idiopathic/isolated rapid eye movement (REM) sleep behavior disorder (iRBD) is a parasomnia characterized by a loss of skeletal muscle atonia during REM sleep and is recognized as an early manifestation of synucleinopathies including Parkinson’s disease (PD) and dementia with Lewy bodies (DLB) [1, 2]. Some studies reported phenoconversion towards an overt neurodegenerative syndrome in 73.5% of patients with iRBD after a 12-year follow-up and in as much as 91% of patients after 14 years [2, 3]. This time window provides an opportunity to identify biomarkers capable of detecting patients with iRBD who are at greater risk of phenoconversion with the goal of implementing neuroprotective trials.

The sensitivity of resting-state electroencephalogram (EEG) assessments to detect pathological changes in iRBD has been demonstrated in several studies. For instance, as compared to healthy controls, studies reported that patients with iRBD exhibit higher theta power in frontal, temporal, and occipital regions as well as lower beta power in the occipital region [4, 5], although other studies also showed lower alpha and beta power in central [6] and higher alpha power in frontal regions [7]. It was also shown that patients with iRBD exhibiting mild cognitive impairment (MCI) show higher theta relative spectral power in posterior regions as opposed to those who are cognitively normal [8]. Furthermore, only a few longitudinal studies using resting-state EEG investigated whether spectral power at baseline can predict cognitive decline or phenoconversion in patients with iRBD [9–11]. A study found higher delta and theta spectral power in central and occipital regions as well as lower beta power in central regions at baseline in patients with iRBD who developed MCI at follow-up [9]. Another longitudinal study reported higher delta and theta relative power in posterior regions as well as reduced beta spectral power in frontal, central, temporal, and occipital regions at baseline in patients with iRBD who converted towards a clinical synucleinopathy as compared to patients who did not convert at follow-up [10]. However, another study found no significant differences between resting-state EEG spectral power at baseline and after a 2-year follow-up in patients with iRBD [11].

The studies mentioned hereabove are based on standard spectral analyses exhibiting a 1/*f*^β^ power law indicative of a *scale-free* arrhythmic component on top of which rhythmic narrowband oscillations take place [12, 13]. Those two cooccurring processes both contribute to the estimation of the spectral power, which can lead to spurious findings in spectral analyses. Hence, narrowband spectral differences are often interpreted as reflecting oscillatory or rhythmic mechanisms, when in fact, they may be linked to changes in the spectral slope of the arrhythmic component [14, 15]. For instance, using the standard procedure for spectral power analysis, a study reported higher theta spectral power in older participants as compared to younger ones, but this difference was abolished after controlling for the arrhythmic component [14, 16]. Thus, recent guidelines argue for the importance of controlling for the arrhythmic component in spectral analysis, especially when investigating potential network dysfunctions [12].

The isolated slope of the arrhythmic component has been found to reflect the balance between excitatory and inhibitory networks, which holds significant relevance in the study of neurodegenerative diseases [17, 18]. Consequently, steeper slopes of the arrhythmic component were identified as sensitive markers of network dysfunction in individuals with DLB and PD, distinguishing them from participants with MCI and healthy participants [19]. Furthermore, another study reported that patients with PD exhibited higher scaling exponents, which translates to steeper slopes of the arrhythmic component, as compared to healthy controls [20]. Although no significant changes were reported regarding the resting-state EEG arrhythmic component after a 2-year follow-up in patients with iRBD [11], no study has yet conducted analyses segregating EEG rhythmic and arrhythmic components in patients with iRBD in relation to phenoconversion.

The ongoing process of synucleinopathies has also been associated with altered integration among distant neural populations (i.e. functional connectivity) [11, 21]. While rhythmic and arrhythmic components indirectly reflect these processes [22, 23], functional connectivity analyses are also needed to directly assess the integrity of large-scale networks. Phase-based functional connectivity measures such as the weighted phase-lag index (wPLI) provide insights into network integrity by examining the consistency of phase-lags between two timeseries [24]. A longitudinal study reported reduced global alpha wPLI at baseline in patients with iRBD when compared to healthy controls [11]. Interestingly, after a 2-year follow-up, these patients demonstrated a significant increase in global alpha wPLI compared to their baseline recording, interpreted by the authors as a compensatory mechanism to counteract the cognitive decline associated with synucleinopathy progression [11].

The aim of this longitudinal study was to assess differences in resting-state EEG measures at baseline to identify markers in patients with iRBD who converted towards a synucleinopathy at follow-up. These measures included relative standard spectral power, rhythmic and arrhythmic components of the power spectra as well as global and pairwise EEG functional connectivity. We hypothesized that at baseline, patients who did convert at follow-up would display higher theta relative standard spectral power as reported in previous work [9, 10]. Upon the factorization of the power spectra, we anticipated that patients who did convert at follow-up would also exhibit steeper slopes of the arrhythmic component, along with higher delta and theta rhythmic power at baseline as reported in patients with DLB and PD [19, 20]. Moreover, we predicted that as compared to patients who did not convert at follow-up, patients who converted would demonstrate lower global and pairwise wPLI at baseline, indicative of an altered functional connectivity in large-scale networks at resting-state as reported in a recent work [11]. Finally, we conducted exploratory analyses to assess potential differences at baseline between iRBD patients who converted towards PD and those who converted towards DLB.

## Materials and Methods

### Participants

Participants who met the International Classification of Sleep Disorders (third edition) criteria for an iRBD diagnosis were recruited for a large longitudinal study at the Center for Advanced Research in Sleep Medicine (CIUSSS du Nord de l’Île-de-Montréal). Participants from this cohort were selected if they had a baseline resting-state EEG recording and at least one neurologic follow-up examination. Additional inclusion criteria included the absence of parkinsonism or dementia at baseline and at least nine of the 10 electrodes of interest (i.e. F3, F4, C3, C4, P3, P4, T3, T4, O1, and O2). Patients were excluded if they were younger than 45 or older than 90 years old at baseline, or if the diagnosed RBD was symptomatic/secondary. The protocol was approved by the ethics committee, and informed consent was obtained from every participant.

### Procedure

The detailed procedure was published in a previous work [10]. The night preceding the baseline resting-state EEG recording, participants underwent a night of video-polysomnography (vPSG) to confirm or rule out iRBD according to the criteria of the International Classification of Sleep Disorders (third edition), which includes loss of REM sleep muscle atonia or excessive phasic activity with further addition of motor activity during REM sleep or clinical history of abnormal motor activity during REM sleep. At baseline and follow-up, participants underwent a comprehensive neuropsychological assessment (JFG) and a neurological examination (RBP), which included the evaluation of depression, anxiety, sleepiness, and the assessment of motor dysfunction. Participants completed the Mini-Mental State Examination scores, the Beck Depression Inventory second edition (BDI-II), the Beck Anxiety Inventory, and the Epworth Sleepiness Scale. The data from the neuropsychological evaluation at baseline was interpreted by a neuropsychologist (JFG) to determine which patient had MCI at baseline with the goal of evaluating if the groups were equivalent regarding the proportion of patients having a MCI. Motor functions were evaluated with the 3^rd^ edition of the Movement Disorder Society’s Unified Parkinson’s Disease Rating Scale (MDS-UPDRS-III). Of note, some data derived from questionnaires and clinical assessments were missing (Supplementary Table S1 for missing data). Following the vPSG recording, participants underwent a resting-state EEG recording 30 minutes after awakening to avoid sleep inertia. Recordings lasted for at least 10 minutes during which a trained technician instructed participants to alternate between eyes-opened and eyes-closed to avoid drowsiness. Annual follow-up examinations were conducted by a neurologist (RBP) to assess whether phenoconversion to parkinsonism or dementia had occurred, according to standard criteria [25, 26]. Upon a diagnosis of synucleinopathy, participants were considered as *converters* for the purpose of this study. Conversely, if participants did not show signs of a neurodegenerative disease at their last neurological evaluation, they were considered as *non-converters*. Detailed information about the neurological and neuropsychological assessments is available in other published works [8, 10]. Briefly, patients were diagnosed with a MCI if (1) a close relative or they themselves reported a subjective cognitive complaint in a structured interview, (2) they showed impairment in at least two variables of the same cognitive domain reflected by a score greater than 1.5 standard deviations below the standardized mean, (3) the cognitive decline was not better explained by a substance use, a psychiatric or a neurologic condition, and (4) they were still able to perform daily activities.

### Data acquisition and preprocessing

Sleep variables were extracted from the vPSG assessment performed on the night preceding the baseline resting-state EEG recording to ensure no significant differences between the groups. These variables included the apnea–hypopnea index, the microarousal index, sleep efficiency, wakefulness after sleep onset, and the percentage of slow-wave sleep. The resting-state EEG assessment was recorded on a Grass polygraph amplifier system (0.3–100 Hz band-pass), and data were digitalized at a 256 Hz sampling rate on a commercially available software (Harmonie, Stellate systems). The EEG montage was based on the international 10–20 system with the ground electrode positioned at the mid-sagittal prefrontal site, and the reference based on the averaged earlobe electrodes. A trained technician selected 96 seconds of eyes-closed sections per participant that were free from cardiac, muscular, or blinking artifacts, and drowsiness signs. However, seven participants had less than 96 seconds of clean EEG segments (minimum recording duration included in the study: 38 seconds). Furthermore, electrodes showing excessive artifacts or noise throughout the full recording were excluded from the analysis and considered as missing channels (Supplementary Table S2 for technical information about epochs and electrodes). Raw recordings were then imported in MATLAB (MathWorks R2022) using custom scripts to segmentalize data in 4-second epochs (Supplementary Table S2).

### Spectral power analyses

#### Relative spectral power.

For each non-overlapping 4-second epoch, Fast Fourier transform was computed using a taper window at a 0.25 Hz spectral resolution and the values were then averaged for each participant. Relative spectral power was derived from the absolute spectral power for each available electrode as a percentage of the whole analyzed power spectrum (i.e. 0.5–32 Hz). The spectral bands defined in this study were delta (0.5–4 Hz), theta (4–7 Hz), alpha (7–13 Hz), beta1 (13–22 Hz), and beta2 (22–32 Hz).

#### Arrhythmic component and rhythmic spectral power.

Several novel methods have been developed to estimate rhythmic and arrhythmic components of the EEG power spectrum [12, 27]. In the present study, we followed a multiplicative model of the spectral power [28] applied to each *n* artifact-free epoch written as


$$\begin{array}{l} p_{i}(\;f)=\;\frac{c_{i}}{f^{\beta_{i}\;}}\;e^{r_{i}\left(\;f\right)},i=1,2,\ldots ,\;n \end{array}$$

where the spectral power *p*_*i*_(*f*) is the product of an arrhythmic component $\frac{c_{i}}{f^{\beta_{i}\;}}$, in which *c*_*i*_ represents an offset value and $f^{-\beta_{i}\;}$, an exponential decay with frequency *f*, multiplying a residual power $e^{r_{i}\left(\;f\right)}$ at frequency *f*. In the absence of rhythmic processes, r_i_(*f*) = 0, and the spectrum would describe a pure arrhythmic epoch. When both axes are log-transformed, most of the spectrum exhibits a dominant wideband linear pattern. For each epoch, this obvious spectral organization does not favorize any narrowband oscillatory mode, but rather reveals a «temporal scale-free» regime modeled with a power law of the form $1/f^{\beta_{i}}$. Up to a minus sign, the exponent β_i_ is the slope of the spectra, which is referred to here as the arrhythmic component of the signal. This slope, and consequently the exponent, may change across epochs. Given this “dynamical” scale-free property across epochs of an interval, we estimated the offset $c_{i}^{⁎}$ and the scaling exponent $\beta_{i}^{⁎}$ from the linear regression of the power spectrum $p_{i}$ in the log-transformed axes scale. Then, we estimated the “rhythmic component” reflected by the «residual spectral power» R(f) as the following:


$$\begin{array}{l} e^{R^{⁎}\left(\;f\right)}=\;\frac{1}{n_{I}}\;\underset{i\in I}{\sum }\;\frac{f^{\beta_{i}^{⁎}}}{c_{i}^{⁎}}\;p_{i}\;(\;f) \end{array}$$

where the exponential of the residual power $e^{R^{⁎}\left(\;f\right)}$ at a given frequency *f* is obtained by the averaged whitened spectrum across epochs in I $\left(\frac{1}{n_{I}}\;\underset{i\in I}{\sum }\;\right)$, in which the estimated offset $c_{i}^{⁎}$ and the scaling exponent $\beta_{i}^{⁎}$ values were obtained from the spectral power $p_{i}\;(\;f)$ of each epoch. This “residual spectral power” was well-suited for frequencies above 4 Hz, but low frequencies were impacted by the high-pass filter of the recording device and consequently, the dynamics in the delta band were not captured by the procedure mentioned hereabove. We thus computed the rhythmic spectral power in the delta band separately by estimating the arhythmic component in this spectral band to isolate the rhythmic component.

### Functional connectivity

The functional connectivity was assessed using the wPLI, which is an index of the phase-lag consistency between two electrodes, and that is robust to volume conduction effects and other sources of noise by weighting down phase-lags of 0 or π [24]. First, the instantaneous phase of the band-pass-filtered analytical signal at each electrode was extracted using the Hilbert transform. Then, for each epoch, the wPLI was computed between every pair of electrodes (*n* = 45), at each frequency *f* with the following definition:


$$\begin{array}{l} wPLI=\;\frac{\left|E\left[I\left(\;f\right)\right]\right|}{E\left[|I\left(\;f\right)|\right]}=\;\frac{\left|E\left[\left|I\left(\;f\right)\right|sng\left(I\left(\;f\right)\right])\right]\right|}{E\left[|I\left(\;f\right)|\right]} \end{array}$$

where I(f) represents the imaginary part of the cross-spectrum at frequency f between two electrodes, and *E*[*I*(*f*)] represents the expectation over the total number of epochs. After this common procedure, the analyses were separated between the global and the pairwise wPLI. The global wPLI represents an index of the connectivity strength that is not specific, but instead gives insight into the integrity of information transfer and integration across the whole surface of the scalp [29]. For this global index of connectivity, the wPLI between each pair of electrodes (*n* = 45) was averaged per 2 Hz mini-bands ranging from 0.5 Hz to 32 Hz to obtain the global wPLI per participant for each mini-band. As for the pairwise wPLI, the predefined bands were the same as used in the spectral power analyses, and the statistical analyses were conducted between every possible pair of electrodes.

### Statistical analyses

All statistical analyses were conducted using MATLAB (MathWorks R2022) custom scripts or Statistical Package for Social Sciences (IBM, 2022). Demographic, neuropsychological, and clinical data were compared between groups using Student’s *t*-tests or Pearson’s Chi-square tests, depending on the nature of the variables and tests’ assumptions, and statistical significance. An important assumption of parametric tests such as the Student *t*-test is the normality of distribution, which was confirmed by a nonsignificant Shapiro–Wilk statistic. In cases of significant Shapiro–Wilk statistics, between-group comparisons were conducted using a non-parametric test. Analyses focusing on EEG variables were performed using a non-parametric test with a pixel-based correction for multiple comparisons [30]. Briefly, participants were randomly shuffled into two temporary groups disregarding original group labels. Then, Student’s *t*-tests were computed for each measured variable of the test after which the minimum and maximum *t*-values (i.e. max T) were extracted and stored in a matrix. This iterative process was repeated 1500 times to compute a null distribution of possible extreme *t*-values observed when ignoring group labels. Comparisons between real groups were calculated for each variable of interest, and the t-values of each test were compared to the distribution of permuted *t*-values to assess significance relative to the max T observed in the permutation distribution. An individual permutation test was computed for each of the five spectral bands for relative and residual spectral power as well as for the pairwise wPLI. The non-parametric test allows to compute a data-driven null-distribution free of prespecified parameters and to assess the significance of group differences based on this null hypothesis [31]. This approach enables to control for familywise error due to multiple comparisons between different electrodes. We chose to report statistical trends and discuss them qualitatively to provide a wider interpretation framework of our results and compare them with previously published works [32]. A two-tailed significance was used for all non-parametric analyses, which corresponds to the 2.5th and 97.5th percentile of each permuted distribution to define a significance threshold of *p* < .05. Statistical trends were defined as results exhibiting a *p*-value higher or equal to 0.05 and lower than 0.10.

## Results

### Sociodemographic, clinical, and PSG variables

Eighteen participants were excluded from the study due to one or more of the following reasons: dementia or a neurological disorder at baseline (*n* = 9), no follow-up evaluation (*n* = 4), younger than 45 years old (*n* = 3), RBD triggered by an antidepression medication acting on serotoninergic neurotransmission (*n* = 1) and more than one missing electrode from the defined montage (*n* = 1). This led to a sample of 81 participants with iRBD (66.89 ± 6.91 [45–90] years old, 61M/20F). After a mean follow-up of 5.01 ± 2.76 years, 34 participants (24M/10F) were diagnosed with an overt neurodegenerative syndrome (follow-up: 3.9 ± 2.4 years before diagnosis with a minimum of 210 days), whereas 47 (37M/10F) remained disease-free (follow-up: 5.9 ± 2.8). Among those in the converter group, 22 were diagnosed with PD and 12 with DLB. At baseline, no significant differences were found between patients who converted and patients who did not convert for age, sex, education, cognitive status, Mini-Mental State Examination scores, BDI-II, Beck Anxiety Inventory, and iRBD symptom duration at baseline (Table 1). Among the sleep variables known to affect EEG measures at wakefulness, we found no significant differences between groups at baseline regarding apnea–hypopnea index, microarousal index, sleep efficiency, wakefulness after sleep onset, and the percentage of slow wave sleep (Table 1). Furthermore, no significant differences were found between groups for anxiolytics and antidepressant intake. Finally, we found that at baseline, patients who converted exhibited significantly higher scores on the MDS-UPDRS-III than patients who did not convert (Table 1).

**Table 1.** Demographic and vPSG Data Between iRBD and Converters

Variable	Non-converters (*n* = 47)	Converters (*n* = 34)	Significance
Age	65.53 ± 7.09	67.81 ± 7.34	*p* = .158
Sex (M/F)	37/10	24/10	*p = *.402
Education	13.68 ± 3.78	12.71 ± 3.83	*p* = .258
MCI, *n* (%)	16 (34)	13 (38)	*p* = .574
MMSE	28.19 ± 1.62	27.69 ± 2.06	*p* = .294
AHI	8.96 ± 11.85	12.04 ± 18.97	*p* = .386
Sleep efficacy (%)	82.30 ± 9.51	82.76 ± 11.31	*p = *.496
Wakefulness after sleep onset (WASO)	81.62 ± 43.02	79.92 ± 49.58	*p = *.523
Slow wave sleep (SWS; %)	6.78 ± 8.29	7.85 ± 9.14	*p = *.669
Micro-arousal index (event/hr)	13.58 ± 7.98	12.47 ± 9.07	*p* = .569
Follow-up (years)*	5.91 ± 2.77	3.88 ± 2.35	*p* < .001*
iRBD symptoms duration	9.32 ± 9.24	7.70 ± 9.35	*p = *.512
BDI	10.15 ± 5.79	8.09 ± 6.49	*p = *.206
BAI	7.45 ± 6.47	6.52 ± 7.06	*p = *.613
ESS	8.28 ± 4.18	7.50 ± 4.81	*p* = .519
MDS-UPDRS-III*	3.25 ± 2.99	6.29 ± 3.65	*p < *.05*
Anxiolytics, *n* (%)	9 (23)	10 (27)	*p = *.195
Antidepressants, *n* (%)	8 (20)	3 (11)	*p = *.335

Significant differences are indicated by *.

### Spectral power

#### Relative standard spectral power.

**Figure 1.**
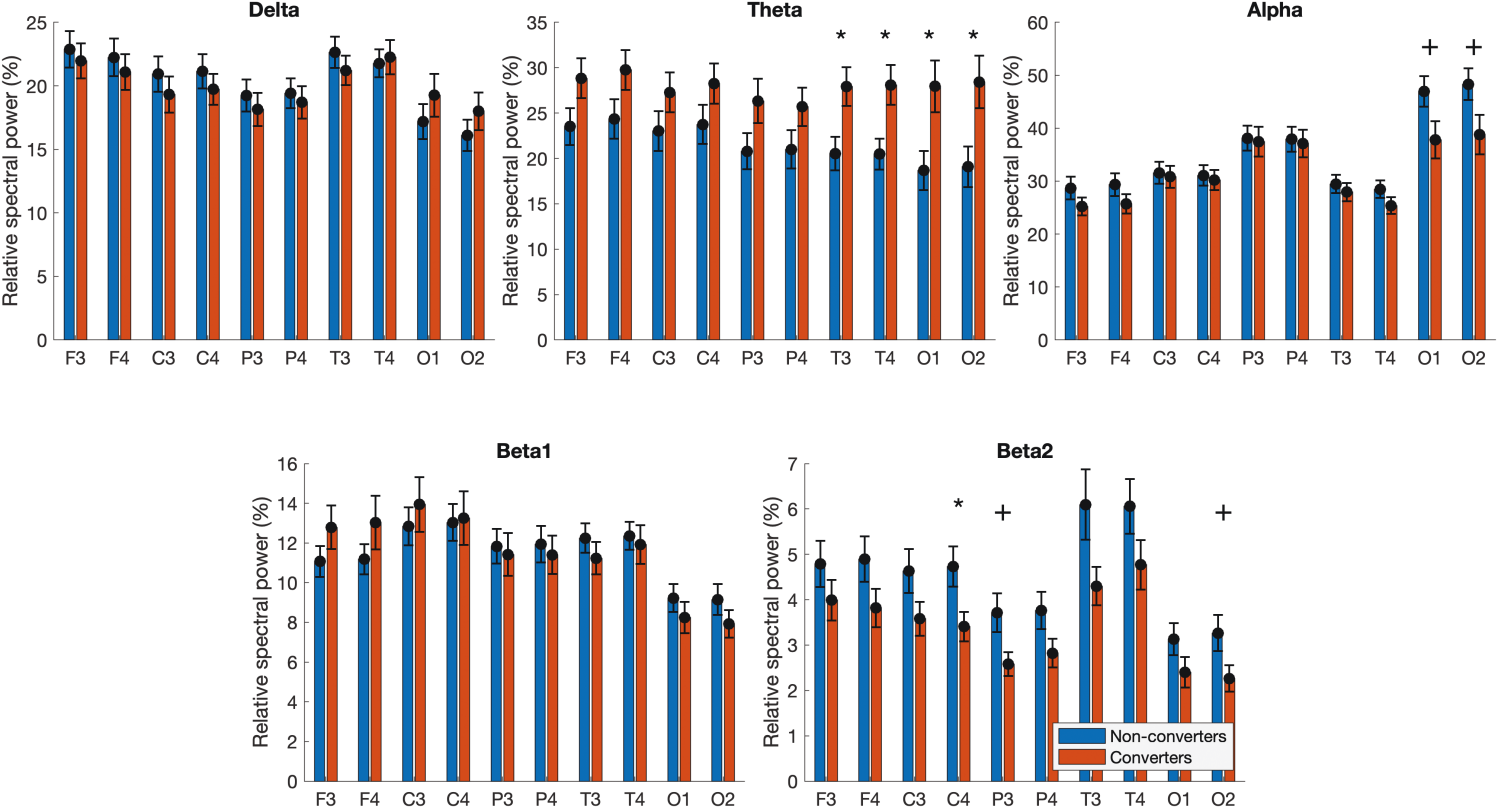
Relative spectral power in iRBD patients who converted (r converters) and who did not convert (non-converters) for each frequency band and electrode. Statistically significant differences are identified by * (*p* < .05) or ** (*p* < .01). Statistical trends are labeled by + (0.05 < *p* < 0.1). Error bars represent standard error.

Significant group differences were found in theta and beta2 spectral bands when using relative standard power analyses (Figure 1). For the theta band, patients who converted exhibited higher relative standard power in T3 (converters: 27.92 ± 2.14; non-converters: 20.53 ± 1.84), T4 (converters: 28.09 ± 2.19; non-converters: 20.49 ± 1.70), O1 (converters: 27.95 ± 2.85; non-converters: 18.67 ± 2.16), and O2 (converters: 28.43 ± 2.88; non-converters: 19.09 ± 2.23). Furthermore, patients who converted exhibited significantly lower beta2 relative standard power in C4 (converters: 3.41 ± 0.33; non-converters: 4.73 ± 0.44). Moreover, statistical trends were found in alpha and beta2. Converters exhibited a trend towards lower alpha standard relative power in O1 (converters: 37.81 ± 3.53; non-converters: 46.95 ± 2.88; *p* = .085) and O2 (converters: 38.79 ± 3.72; non-converters: 48.30 ± 2.98; *p* = .085) when compared to patients who did not convert. As for beta2, patients who converted showed a tendency towards lower relative standard power in P3 (converters: 2.58 ± 0.26; non-converters: 3.71 ± 0.43; *p* = .07) and O2 (converters: 2.26 ± 0.29; non-converters: 3.26 ± 0.40; *p* = .095). No significant effects or trends were found for delta and beta1.

#### Slope of the arrhythmic component.

**Figure 2.**
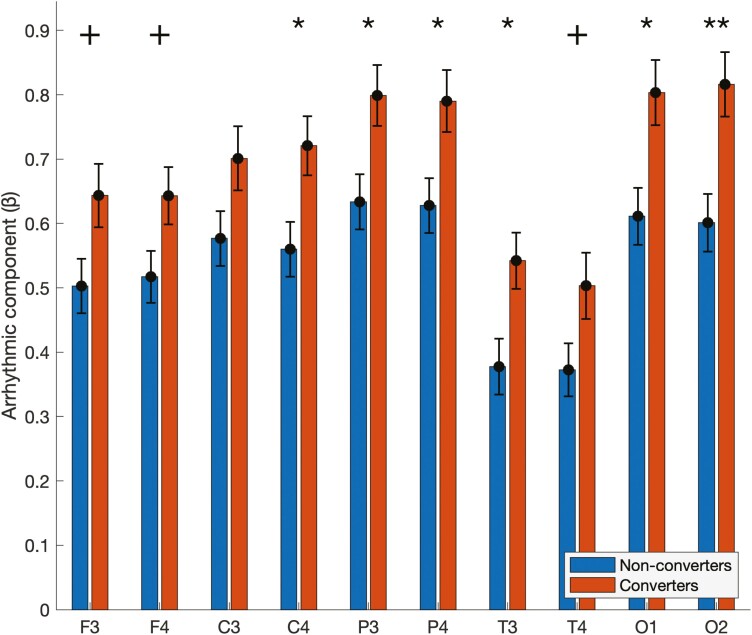
Slope of the arrhythmic component in patients with iRBD who converted (converters) and who did not convert (non-converters) for each electrode. The *y*-axis shows the slope of the arrhythmic component, as represented by the scaling exponent $\beta_{i}$. Statistically significant differences are identified by * (*p* < .05) or ** (*p* < .01). Statistical trends are labeled by + (0.05 < *p* < 0.1). Error bars represent standard error.

At baseline, there was a general pattern of higher scaling exponent values for patients who converted as compared to patients who did not convert (Figure 2). The pixel-based corrected permutation test revealed that patients who converted exhibited significantly steeper slopes in C4 (converters: 0.72 ± .046; non-converters: 0.56 ± .04; *p* < .05), P3 (converters: 0.80 ± .05; non-converters: 0.63 ± .04; *p* < .05), P4 (converters: 0.79 ± .05; non-converters: 0.63 ± .04; *p* < .05), T3 (converters: 0.54 ± .04; non-converters: 0.38 ± .04; *p* < .05), O1 (converters: 0.80 ± .05; non-converters: 0.61 ± .04; *p* < .05), and O2 (converters: 0.82 ± .05; non-converters: 0.60 ± .04; *p* < .01). Furthermore, similar trends were found in F3 (converters: 0.64 ± .05; non-converters: 0.50 ± .04; *p* = .06), F4 (converters: 0.64 ± .04; non-converters: 0.52 ± .04; *p* = .07), and T4 (converters: 0.50 ± .05; non-converters: 0.37 ± .04; *p* = .08).

#### Residual rhythmic spectral power.

**Figure 3.**
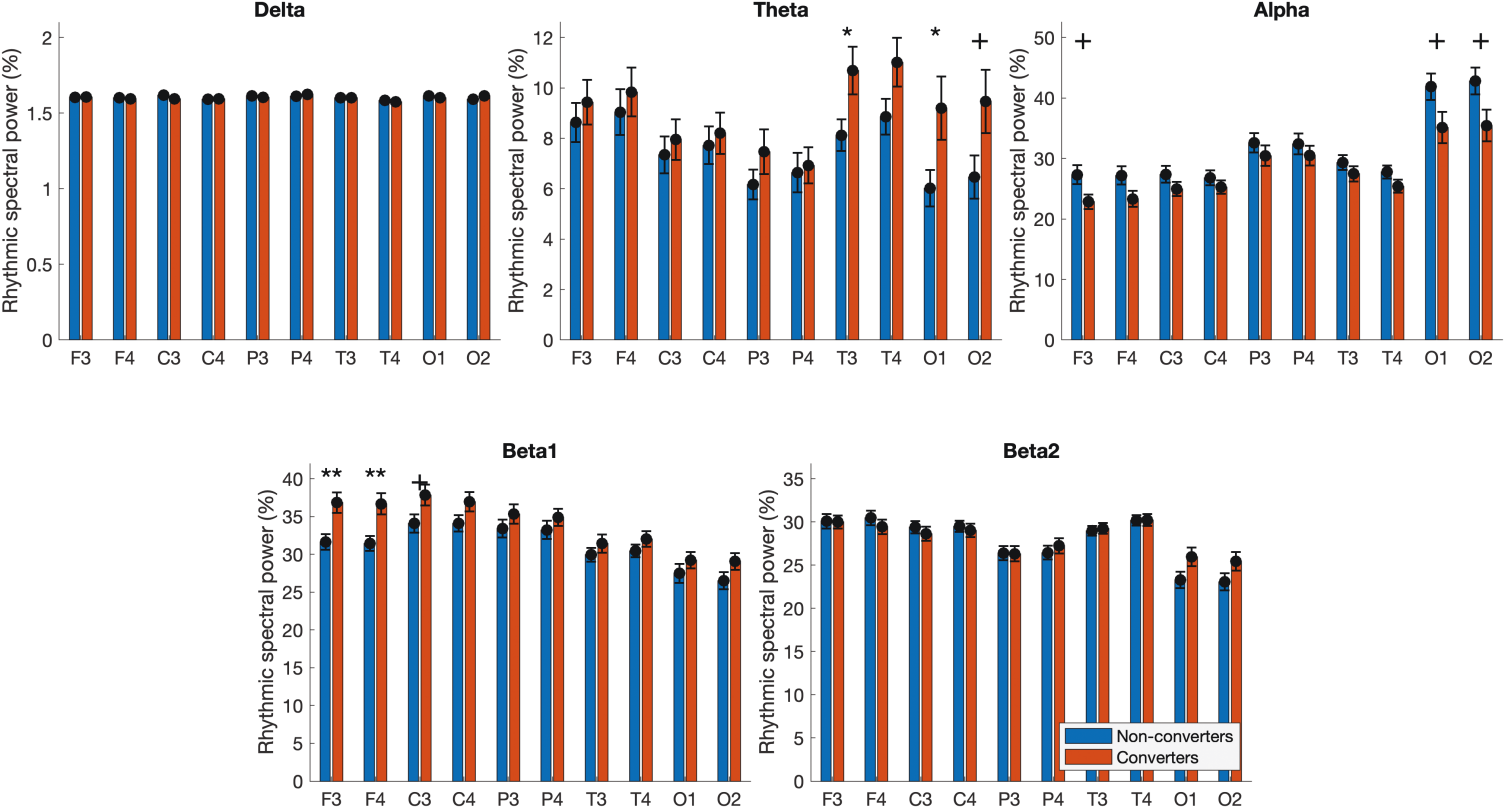
Residual spectral power in iRBD patients who converted (converters) and who did not convert (non-converters) for each frequency band and each electrode. For each spectral band, the residual spectral power (i.e. the rhythmic component) is represented as a percentage of the total residual power after the parametrization of the arrhythmic component for every electrode of the EEG montage. Statistically significant differences are identified by * (*p* < .05) or ** (*p* < .01). Statistical trends are labeled by + (0.05 < *p* < 0.1).

The factorization of the power spectra allowed us to estimate the residual rhythmic spectral power by removing the contribution of the broadband arrhythmic component. Significant differences were found for rhythmic theta and beta1 power (Figure 3). Patients who converted exhibited significantly higher theta rhythmic power in T3 (converters: 10.90 ± 0.94; non-converters: 8.12 ± 0.63, *p* < .05) and O1 (converters: 9.47 ± 1.25; non-converters: 6.03 ± 0.73, *p* < .05). Although not reaching significance, patients who converted showed a trend towards lower alpha rhythmic power in F3 (converters: 22.79 ± 1.20; non-converters: 27.32 ± 1.56; *p = *.07), O1 (converters: 35.21 ± 2.56; non-converters: 41.86 ± 2.19, *p* = .085) and O2 (converters: 35.76 ± 2.60; non-converters: 42.82 ± 2.23, *p* = .06). Patients who converted also exhibited significantly more beta1 rhythmic power in frontal regions (F3: converters: 36.72 ± 1.36; non-converters: 31.66 ± 1.03, *p* < .05; F4: converters: 36.43 ± 1.41; non-converters: 31.43 ± 0.98, *p* < .01). Additionally, patients who converted showed a trend towards higher beta1 rhythmic power in C3 (converters: 37.80 ± 1.38; non-converters: 34.07 ± 1.20; *p* = .09) when compared to patients who did not convert. No other statistical differences or trends were found between the two groups.

### Functional connectivity

#### Global weighted phase-lag index.

**Figure 4.**
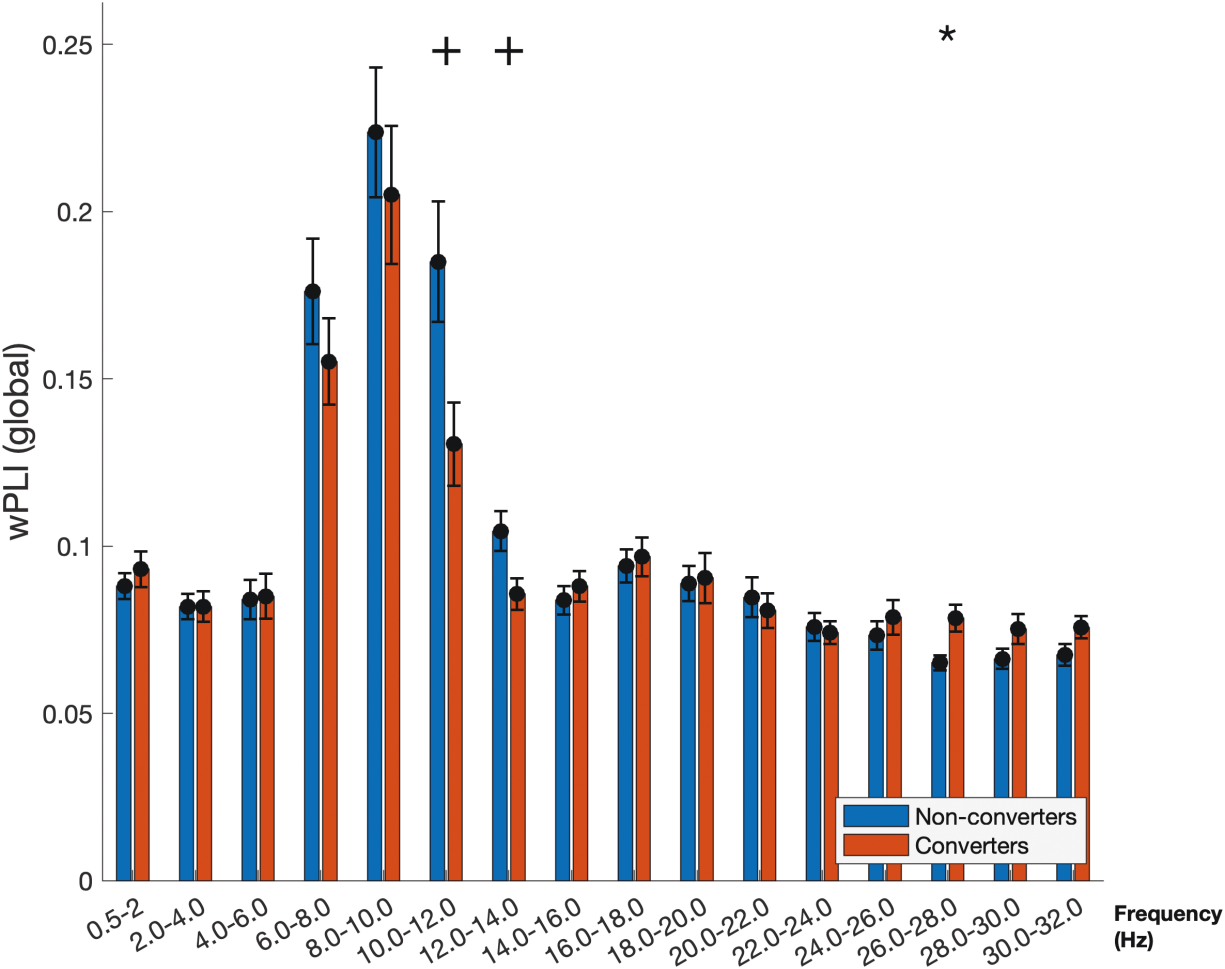
Global weighted phase-lag index in iRBD patients who converted (converted) and who did not convert (non-converters) for frequency bin. The global wPLI (i.e. mean wPLI value between every electrode of the EEG montage) is shown for two Hz mini-bands. Statistically significant differences are identified by * (*p* < .05) or ** (*p* < .01). Statistical trends are labeled by + (0.05 < *p* < 0.1).

When pooling all channels together to assess the global connectivity strength, patients who converted exhibited higher wPLI in the 26–28 Hz mini-band when compared to patients who did not convert (converters: 0.08 ± .004; non-converters: 0.07 ± .002; *p* < .05; Figure 4). Furthermore, the investigation of statistical trends revealed that patients who converted had lower global wPLI in the 10–12 Hz (converters: 0.13 ± 0.01; non-converters: 0.19 ± 0.02; *p* = .075) and 12–14 Hz (converters: 0.09 ± 0.004; non-converters: 0.10 ± .006; *p* = .06) mini-bands when compared to patients who did not convert. No other statistical differences or trends were found.

#### Pairwise weighted phase-lag index.

**Figure 5.**
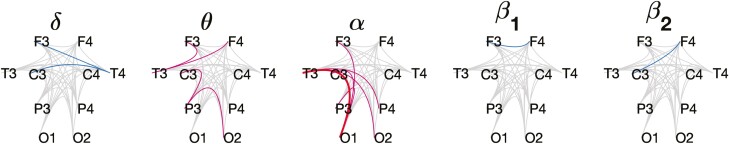
Pairwise weighted phase-lag index across the frequency bands and between all pairs of electrodes. Thin colored lines identify significant differences before correcting for multiple comparisons (*p* < .05, uncorrected), where blue lines represent higher wPLI and pink lines show lower wPLI in converters as compared to non-converters. Thick lines highlight a significant difference between converters and non-converters after applying the pixel-based corrected permutation test with the colors following the same directionality as thin lines (*p* < .05, corrected).

To gain insight into the specific connections affected early on in synucleinopathies, we then computed the wPLI between every pair of electrodes for the five spectral bands of interest. Patients who converted showed significantly lower alpha wPLI between channels T3 and O1 (converters: 0.14 ± .016; iRBD: 0.24 ± .026, *p* < .05) when compared to patients who did not convert (Figure 5). Interestingly, before correcting for multiple comparisons, patients who converted exhibited higher delta, beta1, and beta2 wPLI predominantly between channels located in anterior regions, whereas they appeared to show a widespread lower theta and alpha wPLI in the left hemisphere. No other significant differences or trends were found.

### Complementary analyses according to the trajectory of phenoconversion

Several studies showed the important rate of phenoconversion among patients with iRBD [2, 3, 33], and although it was reported that the proportion of patients phenoconverting towards PD or towards DLB is similar, the question remains whether these two groups differ at baseline upon the analyses conducted in the present study. To verify this, we conducted exploratory analyses in which patients who converted to PD (converters-PD) were compared to patients who converted to DLB (converters-DLB). At baseline, patients who converted to DLB showed significantly higher theta rhythmic power in T3 as compared to patients who converted to PD (converters-DLB: 13.83 ± 1.31; converters-PD: 9.23 ± 1.14; Supplementary Figure S1). To further explore whether theta rhythmic power differences between patients who converted and patients who did not convert were driven solely by the patients who converted to DLB at follow-up, we compared patients who converted to PD and patients who did not convert towards a synucleinopathic disease and found no significant differences (Supplementary Figure S2) supporting the notion that patients who converted to DLB were driving the effect of rhythmic theta. Analyses of the relative standard power, slope of the arrhythmic component, global wPLI, and pairwise wPLI yielded no other significant differences between patients who converted to PD and patients who converted to DLB.

## Discussion

The aim of this study was to assess resting-state EEG components and functional connectivity to identify patients with iRBD at baseline with a higher risk of developing a synucleinopathy. The findings indicate that patients with iRBD who converted exhibited higher theta relative standard power in T3, T4, O1, and O2 and lower beta2 relative standard power in C4, P3, and O2 at baseline as compared to patients with iRBD who did not convert. Additionally, as compared to patients who did not convert, patients who converted showed steeper slopes of the arrhythmic component, predominantly in posterior regions, and higher theta rhythmic power in left temporal and occipital regions, which was mainly driven by patients who converted to DLB. Patients who converted also showed higher beta1 rhythmic power in frontal regions at baseline as compared to patients who did not convert. Moreover, patients with iRBD who converted displayed higher global connectivity in the 26–28 Hz band and lower alpha wPLI between T3 and O1 when compared to patients who did not convert.

### Converters exhibit higher posterior theta and enhanced frontal beta1 rhythmic power

Our findings support previous work indicating that theta relative standard power in posterior regions is a distinctive feature between patients with iRBD and controls [4, 5, 11], patients with iRBD who converted, and those who did not convert [10], as well as patients with PD combined dementia and patients with PD without dementia [34, 35]. Moreover, patients with iRBD who converted also exhibited lower beta2 relative standard power in C4, P3, and O1 compared to patients who did not convert, consistent with previous reports [4, 7, 9, 10].

While various mechanisms have been proposed to explain the slowing of the resting-state EEG in patients with synucleinopathies [5, 36, 37], previous studies overlooked the ubiquitous arrhythmic component in their spectral analyses, thus limiting neurophysiological explanations [12]. After factoring the power spectra, we found that patients who converted exhibited higher theta rhythmic power in T3 and O1 at baseline as compared to patients who did not convert. We thus provide evidence of the rhythmic nature of the well-reported increase in resting-state EEG theta power in patients with iRBD who will convert towards a synucleinopathy at follow-up [19, 38]. Importantly, our supplementary analyses indicate that higher rhythmic theta power at baseline only characterized the subgroup of patients who converted to DLB at follow-up. This result is consistent with a study showing that patients with DLB show higher theta rhythmic power when compared to patients with PD, MCI, and healthy controls [19]. It is important to note that despite the equivalent proportion of patients who converted and those who did not convert who had MCI at baseline, 83% of patients who converted towards DLB had MCI at baseline as opposed to 23% of those who converted towards PD (Supplementary Table S3). Previous articles have highlighted a connection between resting-state EEG slowing and MCI [2, 5, 6, 8, 39], and this observation may elucidate why the elevated theta rhythmic component predominantly stemmed from the subgroup of patients who eventually converted to DLB. Interestingly, a study previously reported that the removal of the arrhythmic component abolished theta rhythmic power differences at resting state between healthy older and younger individuals [15], which further supports the notion that higher theta rhythmic power during resting state is a specific marker of pathological aging.

In the absence of an overt pathology, the resting-state EEG power spectrum is normally dominated by a peak in the alpha band [40, 41], which has been positively correlated to the activity of brain regions involved in the regulation of the default mode network (DMN), including the brainstem as well as structures within temporal and occipital cortices [42]. Coherently, a recent meta-analysis reported DMN hypoconnectivity in patients having a synucleinopathic disease as compared to healthy participants [43]. Thus, our report of higher theta rhythmic power combined with a trend towards lower occipital alpha rhythmic power in converters suggests a shift of the normal resting-state alpha peak towards the theta band that possibly indicates alterations in key regions regulating the DMN, although this hypothesis should be directly verified.

The removal of the arrhythmic background further unveiled higher beta1 rhythmic power in frontal regions among iRBD patients who converted as compared to those who did not convert at follow-up. Previously published work reported that pathological changes are observed in the *substancia nigra* early on in the synucleinopathic progression, which suggests the neurodegeneration of dopaminergic neurons [44, 45]. These early impairments could lead to a loss of efficiency in dopaminergic neurons stimulating inhibitory cortical GABAergic interneurons via the dopaminergic mesocortical pathway [46]. Interestingly, a recent study using knockout mice lacking NMDA receptors on prefrontal cortex inhibitory neurons reported a wideband power increase in a range of higher frequencies including the gamma band, which is consistent with our report of increased beta1 frontal rhythmic power [22].

### Converters exhibit steeper slopes of the spectral arrhythmic component

We reported that patients who converted exhibited steeper slopes of the arrhythmic component at baseline, particularly in posterior regions. It has been suggested that the arrhythmic component reflects the excitation-to-inhibition (E:I) ratio, with steeper slopes indicating lower E:I ratios [13, 17, 18]. In line with this report, previous studies indicated that early Lewy body accumulation in iRBD and synucleinopathies disrupts glutamatergic neuron activity in the subcoeruleus, leading to overactivation of GABA-mediated networks in the ventromedial medulla, which may result in a lower E:I ratio in these brain regions [1, 13, 47]. As the synucleinopathy progresses rostrally, subcortical hubs are increasingly affected, which causes disorganization in multiple networks considering the multiple dopaminergic projections originating from subcortical structures [45, 47, 48]. As mentioned, studies have shown that the alteration of excitatory networks leads to hypoactivation of cortical interneurons, resulting in increased arrhythmicity caused by a loss of temporal structure within the affected neuronal groups [22]. Interestingly, a recent study reported steeper slopes of the arrhythmic component in patients with DLB and PD as compared to healthy participants and patients with MCI during resting-state EEG, interpreted as a marker of network dysfunction caused by ongoing synucleinopathy [19]. In contrast, this difference is opposite to what is observed in healthy aging as older individuals show a flattening of the arrhythmic spectral slope compared to healthy younger participants [49]. Furthermore, a study reported that the slope of the arrhythmic component does not significantly differ between patients with Alzheimer’s disease and control participants, supporting the specificity of the slope of the arrhythmic component as a resting-state EEG marker in synucleinopathies [50]. Taken together, our results and the studies mentioned above suggest that the differences observed in converters at baseline reflect lower E:I ratio indicative of a more severe progression of the synucleinopathy.

### Patients who converted exhibit both hypo- and hyperconnectivity

While the investigation of the rhythmic and arrhythmic components provides insights into the localized activity of large-scale networks and the global balance between them, it does not directly assess the cortical integration of these networks [13]. In our study, we observed a trend towards lower alpha global wPLI and significantly higher global wPLI in the beta range (26–28 Hz), suggesting disruptions in the synchronization and integration of neural activity across distributed brain regions. A previous study reported lower alpha global wPLI in patients with iRBD as compared to controls, although no difference in beta global wPLI was observed [11]. The disruptions in synucleinopathies have been shown to impair multiple functional networks in fMRI^42^ and resting-state EEG studies [11], resulting in both hyper- and hypoconnectivity among these networks. We propose that global connectivity reflects the activity of different large-scale functional networks integrating information at different frequencies in a stable manner over time. At baseline, the more advanced progression of synucleinopathy may lead to impaired regulation of these networks by subcortical regions.

In the pairwise connectivity assessment, we found lower alpha band connectivity in the left temporo-occipital region in converters as compared to patients who did not convert at follow-up. Our finding supports a recent report of a progressively reduced alpha connectivity in patients with PD after a 5-year follow-up [51] and after a 4-year follow-up [52]. Interestingly, altered alpha functional connectivity in patients with PD seems to be prominently found in posterior regions [52, 53]. Although this marker seems to represent a functional connectivity marker of synucleinopathic progression, the topography varies from one study to another, which might be explained by the heterogeneous pathological trajectory in subcortical structures across patients [48, 54]. Furthermore, converters exhibited lower wPLI predominantly in the left hemisphere, which aligns with previous findings from fMRI [55, 56] and resting-state EEG [21] studies. Considering that 88% of the sample was right-handed (Supplementary Table S4), our results support the hypothesis of extended alterations in the dominant hemisphere [55].

Interestingly, left temporal and occipital channels, which exhibited an altered functional connectivity, also showed higher theta rhythmic power, suggesting a relation between the rhythmic component and pairwise functional connectivity. Similarly, before correcting for multiple comparisons, beta 1 in frontal regions showed both higher functional connectivity and higher rhythmic power, further supporting this potential relation. Consistently, a recent study using a neural mass model showed that oscillatory mechanisms originating from the thalamus are directly linked to cortical functional connectivity patterns [57]. We thus propose that the factorization of the power spectra highlights the activity of localized neuronal populations that are part of related functional networks.

### Future directions

A number of markers have been proposed to distinguish patients with iRBD who will convert towards a synucleinopathic disease from those who will not convert [4–11, 29, 35, 58–60]. However, these markers still lack the sensitivity and specificity to predict the disease outcome at the single-subject level [60]. Even when several markers are considered, machine learning models tend to be overfitted, leading to a reproducibility problem, as these models perform poorly when applied to different cohorts [58–60]. Our results clearly support the notion that advanced analyses of clinical EEG assessments have the potential to significantly contribute to the identification of patients with iRBD at a higher risk of phenoconversion in the short term. Performant and accurate predictive models of phenoconversion in patients with iRBD need to be developed to progress toward clinical trials. To achieve this goal, an international effort is needed to standardize EEG measures and build an open-access longitudinal database combining several cohorts with baseline EEG recordings, as well as neuropsychological and clinical data.

### Limitations

The results reported in this study should be interpreted with caution based on the following limitations. First, the non-parametric statistical test used to compare converter and non-converter groups does not control for confounding variables. Group comparison on scores from the MDS-UPDRS-III assessment yields a significant difference, but considering the continuum of the synucleinopathic progression, this could be seen as a confirmation that the groups were at different stages of the disease. No other significant differences between the patients who converted and those who did not convert were found on confounding variables. Nonetheless, the presence of some missing data in the questionnaires assessing anxiety, depression, and sleepiness must be taken into consideration as participants might have differed regarding these variables. Second, the electrode montage used in our study was relatively small compared to other studies evaluating resting-state EEG in patients with iRBD or synucleinopathy [11, 19, 29]. This methodological decision was driven by the longitudinal nature of our research project and the goal of including as many iRBD patients as possible. Third, it is important to note that some of the patients included in this study have also been included in previous work [5, 8, 10, 35]. Therefore, some of the data on which our hypotheses were based might be correlated with the data used in this study. However, the methodology underlying our research, which consisted of separating rhythmic and arhythmic spectral components as well as measuring the functional connectivity, was distinct from previously published work by our research group. Finally, iRBD is a prodromal stage of synucleinopathies highlighting a continuum between the parasomnia and a diagnosed synucleinopathy. This conceptualization of the disease challenges the binary categorization of participants as patients who converted versus those who did not convert. Moreover, longitudinal studies have reported that most patients with iRBD will develop a synucleinopathy over time [2, 33]. However, the significant difference we observed on the MDS-UPDRS-III at baseline supports the notion that iRBD patients who converted at follow-up already exhibited stronger impairment in motor function and thus formed a distinctive group of patients.

## Conclusion

In conclusion, our study reveals compelling insights into the early stages of synucleinopathic progression in individuals with iRBD who are at a higher risk of phenoconversion. By examining alterations in both rhythmic and arrhythmic components and functional connectivity derived from EEG resting-state activity, we unveil that early on, the pathology leads to dysfunctions in subcortical hubs causing a widespread imbalance between excitatory and inhibitory networks. The present work introduces a novel and noninvasive framework for studying synucleinopathies, with potential applications beyond this specific context. Notably, the separation of rhythmic and arrhythmic components offers a better understanding of neurophysiological mechanisms, shedding light on standard spectral analysis findings. Future studies integrating complementary neuroimaging techniques should establish the neurological correlates of these EEG components, further advancing our understanding of both synucleinopathies and broader neurodegenerative disorders.

## Supplementary material

Supplementary material is available at *SLEEP* online.

zsae074_suppl_Supplementary_Material

## Data Availability

The data underlying this article will be shared on reasonable request to the corresponding author.
