# Supplementary material for: Electroencephalogram rhythmic and arrhythmic spectral components and functional connectivity at resting state may predict the development of synucleinopathies in idiopathic rapid eye movement sleep behavior disorder
Source: Sleep. 2024 Mar 18;47(12):zsae074. doi: 10.1093/sleep/zsae074 (PMC11632188; doi:10.1093/sleep/zsae074)
Supplement: zsae074_suppl_Supplementary_Material [file zsae074_suppl_supplementary_material.docx]

**Supplementary material**

**Title**: EEG rhythmic and arrhythmic spectral components and functional connectivity at resting state may predict the development of synucleinopathies in idiopathic REM sleep behavior disorder

**Authors:** Hernandez, J.^1,2^, Lina, J.-M.^1,6^, Dubé, J.^1,3^, Lafrenière, A.^1,3^, Gagnon, J.-F.^1,4^, Montplaisir, J.-Y.^1,7^, Postuma, R. B.^1,5^, Carrier, J.^1,3^

**Affiliations :**

1. Center for Advanced Research in Sleep Medicine, Research center, CIUSSS du Nord de l’Île-de-Montréal, Montreal, Canada.
2. Department of Neuroscience, Université de Montréal, Montreal, Canada.
3. Department of Psychology, Université de Montréal, Montreal, Canada.
4. Department of Psychology, Université du Québec à Montréal, Montreal, Canada.
5. Department of Neurology and Neurosurgery, Montreal Neurological Institute, Montreal, Canada.
6. École de technologie supérieure, Montreal Canada.
7. Department of psychiatry, Université de Montréal, Montreal, Canada

Corresponding authors :

Julie Carrier, PhD.

Jimmy Hernandez, MSc

Center for Advanced Research in Sleep Medicine, Hôpital du Sacré-Coeur de Montréal, 5400 boul. Gouin Ouest, Montreal , Quebec Canada, H4J 1C5

[Julie.carrier.1@umontreal.ca](mailto:Julie.carrier.1@umontreal.ca)

[Jimmy.hernandez@umontreal.ca](mailto:Jimmy.hernandez@umontreal.ca)

1. **Supplementary tables**

|  | Non-converters (n = 47) | | Converters (n = 34) | |
| --- | --- | --- | --- | --- |
|  | **Valid data** | **Missing data** | **Valid data** | **Missing data** |
| Mini-Mental State Examination | 31 | 16 | 29 | 5 |
| Beck Depression Inventory | 39 | 8 | 22 | 12 |
| Beck Anxiety Inventory | 38 | 9 | 21 | 13 |
| Epworth Sleepiness Scale | 38 | 9 | 20 | 14 |

**Supplementary Table 1**: Valid and missing data concerning the questionnaires for non-converters and converters.

|  | Non-converters (n=47) | Converters (n=34) | T-test |
| --- | --- | --- | --- |
| Number of 4s epochs from the resting-state EEG assessment | 26.66 ± 2.22 | 26.26 ± 3.46 | t(79) = -.625, *p* = .533 |
| Electrode F3, valid n (%) | 46 (98) | 33 (97) | - |
| Electrode F4, valid n (%) | 46 (98) | 32 (94) | - |
| Electrode C3, valid n (%) | 45 (96) | 34 (100) | - |
| Electrode C4, valid n (%) | 47 (100) | 33 (97) | - |
| Electrode P3, valid n (%) | 46 (98) | 33 (97) | - |
| Electrode P4, valid n (%) | 47 (100) | 34 (100) | - |
| Electrode T3, valid n (%) | 43 (91) | 34 (100) | - |
| Electrode T4, valid n (%) | 45 (96) | 33 (97) | - |
| Electrode O1, valid n (%) | 47 (100) | 34 (100) | - |
| Electrode O2, valid n (%) | 46 (98) | 34 (100) | - |

**Supplementary Table 2**: Technical information about resting-state EEG recordings. First row indicate the number of 4s epochs included in the analyses for non-converters (center column) and converters (last column). The epochs represent artifact-free sections from the eyes-opened condition of the resting-state EEG assessment. Second to last row indicate the number of valid electrode included in the study for both groups.

|  | Parkinson’s Disease (n=22) | Dementia with Lewy bodies (n=12) |
| --- | --- | --- |
| MCI at baseline, n (%) | 5 (23) | 10 (83) |
| Cognitively normal, n (%) | 17 (77) | 2 (17) |
| Missing data, n (%) | 0 (0) | 0 (0) |

**Supplementary Table 3**: Proportion of patients who had MCI at baseline among the group of iRBD patients who converted towards a synucleinopathic disease at follow-up.

|  | Non-converters (n = 47) | Converters (n = 34) |
| --- | --- | --- |
| Right-handed | 19 | 25 |
| Left-handed | 1 | 1 |
| Both | 3 | 1 |
| Missing data | 24 | 7 |

**Supplementary Table 4**: Handedness between non-converters (center column) and converters (right column). The last row shows missing data for both groups.

1. **Supplementary Figures**


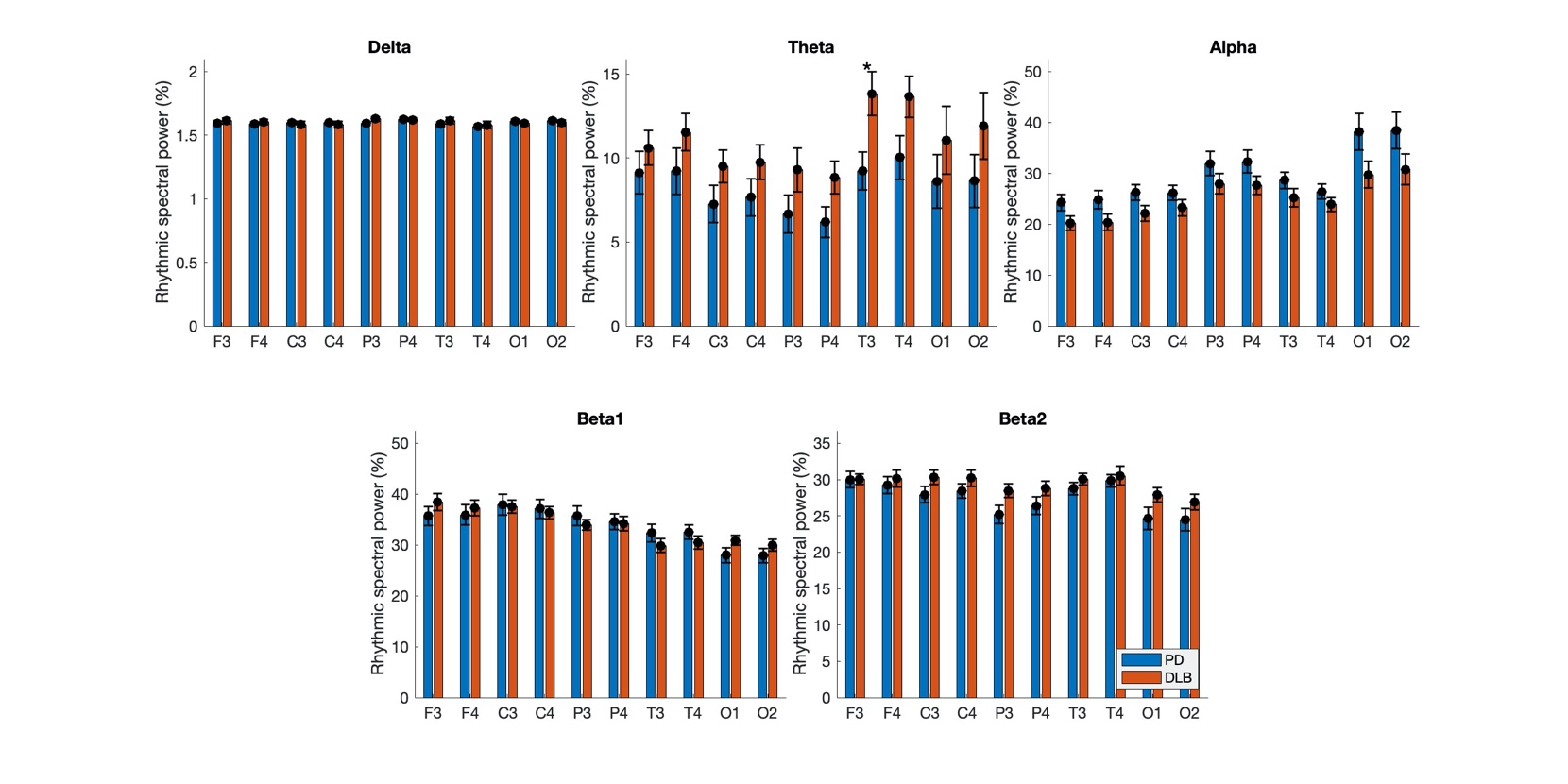


**Supplementary Figure 1:** Residual spectral power in iRBD patients who converted towards Parkinson’s disease (blue bars; PD) and who converted towards dementia with Lewy body (red bars; DLB) for each frequency band and each electrode

For each spectral band, the residual spectral power (i.e., the rhythmic component) is represent as a percentage of the total residual power after the parametrization of the arrhythmic component for every electrodes of the EEG montage. Statistically significant differences are identified by * (p < .05) or ** (p < .01). Error bars represent standard error.


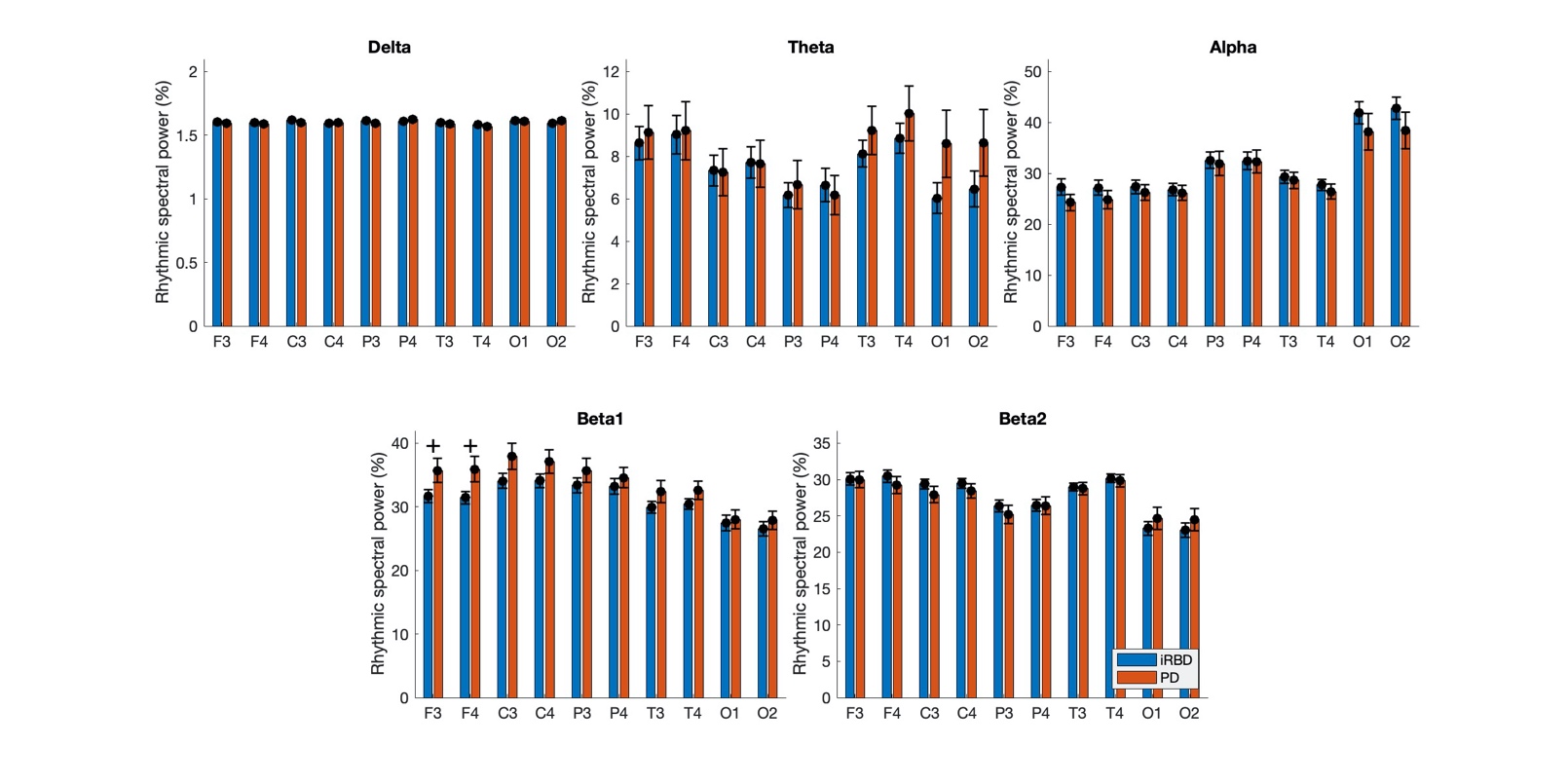


**Supplementary Figure 2:** Residual spectral power in patients with iRBD (blue bars; iRBD) and patients who converted towards Parkinson’s disease (red bars; PD) for each frequency band and each electrode

For each spectral band, the residual spectral power (i.e., the rhythmic component) is represent as a percentage of the total residual power after the parametrization of the arrhythmic component for every electrodes of the EEG montage. Statistically significant differences are identified by * (p < .05) or ** (p < .01). Statistical trends are labeled by + ( 0.05 < p < 0.1). Error bars represent standard error.
